# *ABCG2* polymorphism and rivaroxaban pharmacokinetics in healthy individuals after a single dose

**DOI:** 10.1590/1414-431X2024e13257

**Published:** 2024-07-01

**Authors:** A.F. dos Santos, Q.A.S Francisco, J.B. Nunes, F.A. Colombo, V.B. Boralli

**Affiliations:** 1 Universidade Federal de Alfenas, Alfenas, MG, Brasil; 2 Instituto Claudia Marques de Pesquisa e Desenvolvimento, Pouso Alegre, MG, Brasil

**Keywords:** Polymorphism, Transporter gene, Breast cancer resistance protein, Direct oral anticoagulants, Pharmacokinetics, Variability

## Abstract

Rivaroxaban is a direct factor Xa inhibitor. Its interindividual variability is large and may be connected to the occurrence of adverse drug reactions or drug inefficacy. Pharmacogenetics studies concentrating on the reasons underlying rivaroxaban's inadequate response could help explain the differences in treatment results and medication safety profiles. Against this background, this study evaluated whether polymorphisms in the gene encoding the *ABCG2* transporter modify the pharmacokinetic characteristics of rivaroxaban. A total of 117 healthy volunteers participated in two bioequivalence experiments with a single oral dose of 20 mg rivaroxaban, with one group fasting and the other being fed. Ultra-high-performance liquid chromatography coupled with mass spectrometry was employed to determine the plasma concentrations of rivaroxaban, and the WinNonlin program was used to calculate the pharmacokinetics parameters. In the fasting group, the rivaroxaban pharmacokinetic parameters of Vd (508.27 *vs* 334.45 *vs* 275.59 L) and t1/2 (41.04 *vs* 16.43 *vs* 15.47 h) were significantly higher in *ABCG2* 421 A/A genotype carriers than in *ABCG2* 421 C/C and 421 C/A genotype carriers (P<0.05). The mean values of Cmax (145.81 *vs* 176.27 *vs* 190.19 ng/mL), AUC^0-t^ (1193.81 *vs* 1374.69 *vs* 1570.77 ng/mL·h), and Cl (11.82 *vs* 14.50 *vs* 13.01 mL/h) for these groups were lower, but this difference was not statistically significant (P>0.05). These findings suggested that the *ABCG2* 421 A/A genotype may impact rivaroxaban parameters after a single dose in healthy subjects. This finding must be validated before it is applied in clinical practice.

## Introduction

Rivaroxaban is a direct inhibitor of coagulation factor Xa and is classified as a direct oral anticoagulant (DOAC). It has been approved for the prevention and treatment of thromboembolic disease (1,2). DOACs are currently the most commonly prescribed antithrombotic drugs, and the market is projected to grow further in the coming years. The increased use of anticoagulant treatment during the COVID-19 pandemic also had an impact on this market (3).

The oral bioavailability of rivaroxaban at doses of 10 mg or less is estimated to range from 80 to 100%, and this rate is not altered by food. Oral bioavailability of rivaroxaban at 15 and 20 mg doses was found to be 66%, and co-administration of rivaroxaban with food resulted in high bioavailability (80%) (4,5). Rivaroxaban is rapidly absorbed and reaches maximum plasma concentration (Cmax) within two to four hours of oral administration (4-7). After being absorbed from the lumen of the intestinal tract, rivaroxaban is excreted from the body via multiple pathways, the most common of which being the renal route (66%) followed by the fecal/biliary route (28%). The percentage of the initial dose excreted as an unchanged drug was found to be 43%, with 36% excreted via urine and 7% excreted via feces (6-9). Of the 36% of the rivaroxaban dose eliminated in the urine, approximately 30% is excreted by active renal secretion while the remainder is excreted by glomerular filtration (7-9). P-glycoprotein and breast cancer resistance protein (BCRP, encoded by the *ABCG2* gene) act as transporters in the absorption from the gut lumen and excretion in the renal tubule. In doses of up to 20 mg, a mean terminal half-life of about six to nine hours and a volume of distribution of about 50 L were found in young, healthy subjects (1,3,7-9). Rivaroxaban is a Biopharmaceutics Drug Disposition Classification System (BDDCS) Class 2 drug, whose transport would generally be affected by clinically relevant interactions with poorly soluble and highly metabolized drugs (10).

Drug transporters are found in numerous tissues of the body, which makes them players in drug distribution. They modulate access to metabolizing enzymes and both biliary and renal excretion processes. In cases where the function of drug transporters is altered by genetic polymorphism in the transporter gene, the resulting change in the volume of distribution can lead to a significant change in the effect of the drug or the likelihood of toxicity, as well as a shift in half-life independent of a change in clearance. An efflux transporter with a decreased function increases accumulation in the tissue expressing the transporter. The effect on the total distribution volume depends on the tissue expressing the transporter (11). Polymorphisms in transporter genes may affect rivaroxaban bioavailability and, thus, its safety profile due to its pharmacokinetic characteristics (12-16).

The pharmacokinetics and pharmacodynamics of rivaroxaban were found to be predictable across a wide range of doses in both healthy individuals and patients. Significant interindividual and intraindividual variability of rivaroxaban pharmacokinetics has been documented in various settings, including healthy volunteers, patients enrolled in clinical trials, and real-world cohorts, although the variability is not fully explained (12,17,18). Although several factors may influence this variability, genetic polymorphisms, particularly enzymes involved in transport and metabolism, may play an important role in the pharmacokinetics and pharmacodynamics of various drugs. Polymorphisms in transporter genes may affect rivaroxaban bioavailability and, thus, its safety profile due to its pharmacokinetics (12-14).

Rivaroxaban is a BCRP substrate that has been increasingly recognized as an essential mediator of drug transport in the gut and renal tubules. Although several single nucleotide polymorphisms (SNPs) are present in the *ABCG2* gene, one SNP in particular has raised much interest, the reduced function of which is rs2231142 C421A (Q141K) and the *A* allele is associated with poorer function, i.e., poorer transport of ABCG2 substrate drugs. A genotype associated with decreased expression and function of BCRP protein may predict decreased drug efflux mediated by BCRP, which is associated with increased efficacy but with a risk of increased toxicity (11,15-20).

The International Transporter Consortium has recommended designing and implementing drug-drug interaction studies. In addition, it is recommended that a pharmacogenetic approach be applied to any new molecular entity in which BCRP plays a significant role in the disposition or response due to the high allele frequencies of the reduced function BCRP Q141K variant. This is considered especially significant given the number of BCRP substrates on the World Health Organization list of essential medicines (12,21-23).

The main objective of this study was to assess the pharmacokinetics of rivaroxaban in a group of healthy volunteers and to connect this information to a particular SNP, *ABCG2* c.421C>A, which is found on the *ABCG2* gene.

## Material and Methods

### Study population

A total of 120 individuals consented to participate in two randomized and crossover bioequivalence tests conducted at the Claudia Marques Research and Development Institute (Brazil). The participants were split into two groups: one consisting of 60 individuals who fasted before the study and the other consisting of 60 individuals who were fed before the study. The researchers collected sociodemographic data, including age, gender, weight, height, body mass index (BMI), contraceptive usage, and tobacco consumption. Signed informed consent was obtained from all participants for bioequivalence and pharmacogenetics research. Each individual signed two informed consent forms and was asked to self-report their skin color according to the Brazilian Census classification scheme, which relies on the self-perception of skin color. The approach complied with the current Brazilian guidelines for clinical research involving human participants and received approval from the Research Ethics Committee, following the Guideline of Good Clinical Practice.

The study comprised individuals who met all the specified inclusion criteria and did not meet any exclusion criteria. The eligibility criteria included the recognized standards utilized in bioequivalence studies. Specifically, the criteria for inclusion in the study were volunteers of both genders, aged between 18 and 50 years, without any organic or psychiatric disorders, with typical vital signs and electrocardiogram (ECG) results, with regular medical records and physical examination results, and without any clinically significant abnormalities in hematology, biochemistry, serology, or urine tests. The exclusion criteria consisted of individuals with organic or psychiatric conditions, regular use of medications, BMI outside the range of 18.5-30.0 kg/m^2^, a history of drug allergies, suspicion of consuming controlled substances or alcohol, smoking, pregnant or breastfeeding, and having donated blood within the three months before the start of the trial. Participants had the freedom to withdraw from the study at any point.

### Study design

An open-label, randomized, single-dose, fully replicated (four-periods) design was used in this trial, with two sequences (TRTR or RTRT) consisting of test (T) or reference (R); of two formulations of 20 mg rivaroxaban tablets given orally to healthy volunteers under fasting or fed conditions. Fasting subjects were given a single dose of the test or reference formulation with 200 mL of water following an overnight fast of at least 10 h. The fast continued for four hours after administration. A seven-day washout period took place between each study session. The fed volunteers performed the same protocols and collection schedule as the fasted group, except that they ate a high-fat breakfast of 800 to 1000 kcal. Breakfast was eaten 30 min before administration and was finished in less than 30 min. Blood samples (4.9 mL) were extracted into tubes containing heparin sodium at 0, 0.33, 0.66, 1.0, 1.33; 1.66, 2.0, 2.33, 2.66, 3.0, 3.33, 3.66, 4.0, 4.5, 5.0, 5.5, 6.0, 8.0, 10.0, 12.0, 24.0, 48.0, and 72.0 h after dosing and centrifuged for 10 min at 1900 *g* and 4°C. The plasma samples were separated and kept at about -20°C until analysis.

### Rivaroxaban quantification

Rivaroxaban plasma concentrations were determined at the Instituto Claudia Marques de Pesquisa e Desenvolvimento laboratories using ultra-high-performance liquid chromatography-tandem mass spectrometry (UHPLC-MS/MS). The procedure included a liquid-liquid extraction process. A 25 μL internal standard (linezolid) was added to a 100 μL plasma sample, which was then extracted for three minutes by employing 50 μL of acetonitrile and 1000 μL of ethyl acetate/hexane (80:20 v/v) combined. The supernatant (600 μL) was collected and dried under a compressed air flow. The residue was resuspended in 200 μL ultrapure water and acetonitrile (60:40 v/v) and transferred to a vial with an insert, three μL of which was injected into the chromatographic apparatus. An analytical column (Waters Acquity UPLC BEH, USA, C18, 1.7 m 2.1×100 mm) was used for chromatographic separation. Mobile phase A was ammonium acetate 2 mmol/L in water with 0.025% formic acid, and mobile phase B of acetonitrile was isocratically performed. The analytical pump flow rate was 0.2 mL/min, and the column temperature was maintained at 35°C. A tandem mass spectrometer (Xevo TQ-S/Waters Corp., USA) was used to identify the analyte under positive electrospray ionization conditions. The temperature of the ion source was 150°C. The multiple reaction monitoring transitions for rivaroxaban were 436.2/145.0 (m/z) and 338.2/195.2 (m/z) for the internal standard (IS). The calibration standards for rivaroxaban were validated in the range of 1.0 to 500 ng/mL, with a lower limit of quantification of 1.0 ng/mL. ANVISA resolution (24) was used to validate the method.

### Pharmacokinetic parameters

Pharmacokinetic parameters were calculated using WinNonlin Professional Edition (Version 8.0, Pharsight Corporation, USA). For each of the formulations studied (T and R), descriptive statistical analyses were performed, and means±SD of the parameters were calculated. The area under the concentration-time curve (AUC) was calculated by employing linear trapezoidal integration (AUC^0-72^), and the maximum concentration (Cmax) was obtained directly from the raw data. The terminal half-life (t1/2, h), distribution volume (Vd/F, L), and oral clearance (Cl/F, L/h) were also estimated. The ANVISA-recommended statistical study of the pharmacokinetic bioequivalence of the test formulation was compared to that of the reference formulation. The log-transformed AUCs and Cmax were analyzed using the ANOVA model, with treatment as the main factor. The σWT and σWR deviations were calculated, and the within-subject variability of the T and R formulations was compared, with 2.5 being the top limit of the 90% confidence intervals for the test-to-reference ratio of within-subject variability.

### Pharmacogenetic analysis

The selection of polymorphisms was established by considering the pharmacokinetics of rivaroxaban. *ABCG2*, which codes for the BCRP transporter protein, was chosen as the target gene. Specifically, the polymorphism of interest was the 421C>A variant (rs2231142). Blood samples were collected for DNA extraction using a 4 mL EDTA K2 tube. Subsequently, the samples were kept under refrigeration at a temperature of -20°C. The samples were transported under refrigeration to the Molecular Biology Laboratory at the Federal University of Alfenas in Alfenas, Brazil. DNA extraction was performed using a commercially available kit (PureLink™ Genomic DNA Mini kit, Invitrogen, USA), and the DNA concentration was determined using a spectrophotometer (Jenway Genova Nano, UK). The amplifications were conducted with a commercially available kit (GoTaq^®^ Green Master Mix, Promega, USA) that consisted of two dyes, one of which was blue and the other yellow. These dyes facilitated the monitoring of the samples' progression during electrophoresis. The amplifications were conducted with a thermal cycler (GeneAmp^®^ PCR System 9700, Applied Biosystems, USA). Genetic material was identified with a set of primers. Following gene material amplification, the samples were subsequently forwarded to an external laboratory for sequencing using the automated Sanger technique. The genomic sequences were analyzed and assembled using the BioEdit Sequence Alignment Editor Version 8.1 (https://bioedit.software.informer.com).

### Statistical analysis

Given this study's exploratory nature, a formal sample size calculation was not undertaken. The Shapiro-Wilk normality test was conducted on each group of data. The Student's *t*-test or ANOVA test was performed comparing two or more groups, depending on the normality assumption. Tukey's *post hoc* test was employed to compare pairwise means among different groups. A significance level of P<0.05 indicated statistical significance. The statistical analyses were conducted using Minitab^®^ Statistical Package Version 18.1 (Minitab, Inc., USA).

## Results

### Demographic characteristics

This genetic study included 117 healthy participants who completed two bioequivalence trials. Sixty participants (30 men and 30 women) were included in the fasting condition and 57 volunteers (29 men and 28 women; 3 individuals did not complete the study) were included in the fed condition. Demographic data were comparable among the studies, as shown in Table 1.

**Table 1:** Demographic characteristics of the total study population (n=117).

	Fasting (n=60)	Fed (n=57)
Age (years, mean±SD)	30.55±7.59	32.37±8.16
Weight (kg, mean±SD)	68.51±10.22	70.25±12.16
Height (m, mean±SD)	1.67±0.11	1.68±0.10
BMI (kg/m^2^, mean±SD)	24.53±2.55	24.89±2.91
Gender (n)		
Male	30	29
Female	30	28
Ethnicity^*^ (n)		
White	30	33
Black	8	6
Brown	22	16
Amerindian	0	2
Yellow	0	0

Based on the Brazilian census classification. SD: standard deviation.

### Pharmacokinetic analysis

**Figure 1:**
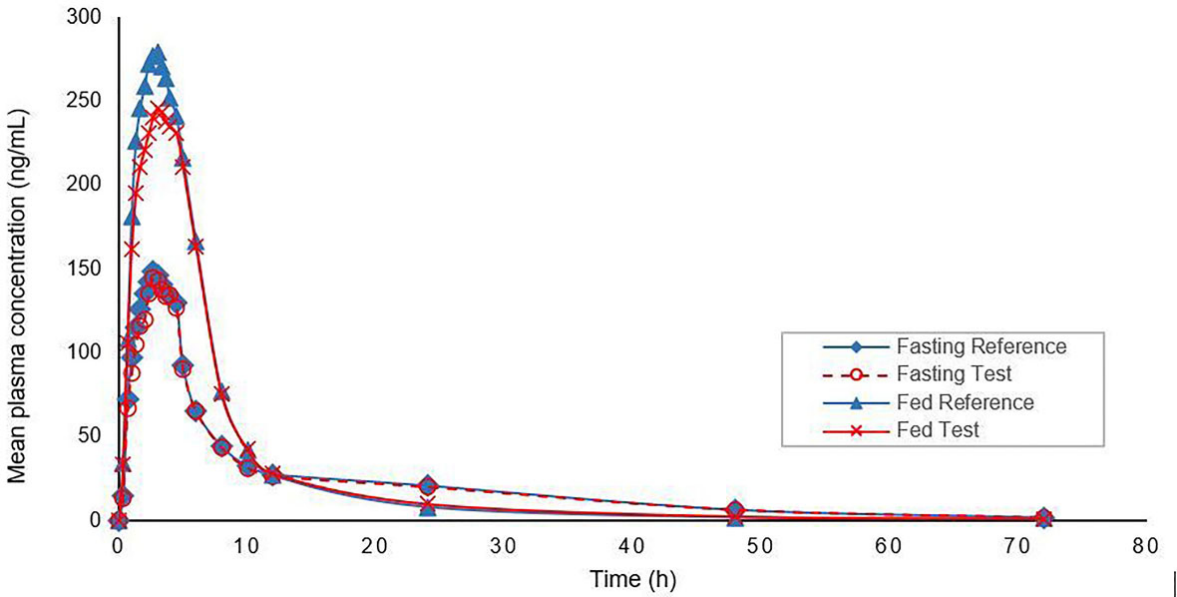
Mean plasma concentrations *vs* time profiles after a single dose of rivaroxaban (20 mg) under fasting (n=60 volunteers) and fed conditions (n=57 volunteers).

Table 2 reports the means and standard deviations of rivaroxaban pharmacokinetic parameters (reference and test formulations) for the total number of participants and according to the condition studied (fasting and fed). The fed group exhibited greater AUC^0-t^, AUC^0-inf^, Cmax, and Tmax values of rivaroxaban than the fasting group, and the mean concentration-time curves are shown in Figure 1. These pharmacokinetic characteristics are consistent with previous research (4,5,25,26).

**Table 2:** Pharmacokinetic parameters of rivaroxaban according to the condition studied (fasting and fed).

Parameters	Fasting (n=240)		Fed (n=228)	
	Reference	Test	Reference	Test
C_max_ (ng/mL)	191.12±6.59	183.98±6.72	325.41±7.32	299.92±7.70
AUC^0-t^ (ng/mL·h)	1573.82±46.21	1509.91±42.11	1969.12±53.73	1888.29±49.99
AUC^0-inf^ (ng/mL·h)	1685.15±49.35	1674.33±53.55	2037.85±66.70	1932.85±50.24
T_max_ (h)	2.26±0.11	2.48±0.12	3.03±0.10	3.10±0.12
t1/2(h)	15.24±0.60	17.57±1.33	8.50±0.41	10.11±0.48
Vd/F (L)	271.73±10.15	304.77±13.26	126.68±6.23	158.95±7.79
Cl/F (mL/h)	13.00±0.35	13.19±0.37	10.73±0.29	11.19±0.31

Data are reported as mean±SD. C_max_: maximum concentration; AUC^0-t^: area under the curve from zero to last determined time; AUC^0-inf^: area under the concentration-versus-time curve from time zero to infinity; T_max_: time corresponding to the occurrence of the maximum plasmatic concentration; t1/2: half-life drug elimination; Cl: clearance; Vd: volume of distribution; F: bioavailability.

### Genotypic frequencies and sequencing

**Figure 2:**
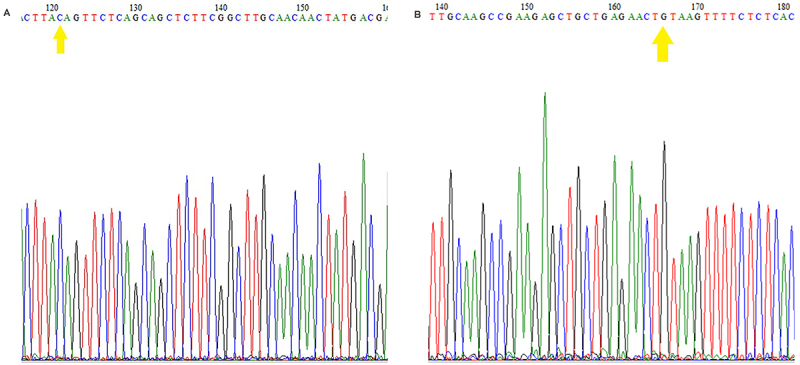
Electropherogram representing the 421C>A polymorphism. Result of sequencing the two strands with forward (**A**) and reverse (**B**) primers from the research participant 01, with 421 C/C wild-type. The yellow arrows indicate nucleotides C (forward primer) and G (reverse primer), representing the 421 C/C: wild-type genotype.

**Figure 3:**
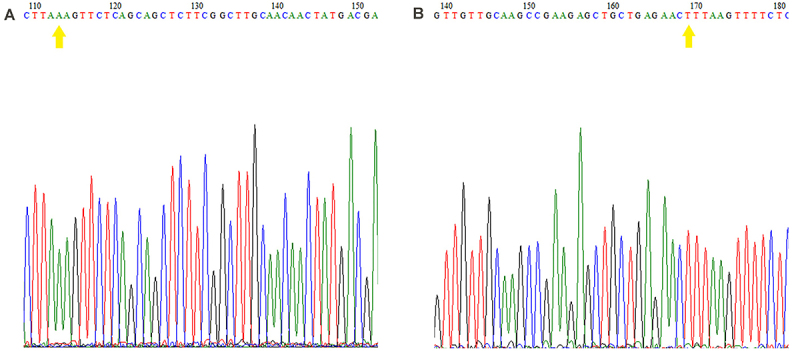
Electropherogram representing the 421C>A polymorphism. Result of sequencing the two strands with forward (**A**) and reverse (**B**) primers from research participant 07, with 421 A/A homozygous mutant. The yellow arrows indicate nucleotides A (forward primer) and T (reverse primer), representing the 421 A/A mutant genotype.

From the results of Sanger sequencing, representative electropherograms of the exon 5 region containing the SNP rs2231142 were obtained. Figures 2 and 3 show the electropherograms of two sequenced homozygous samples (wild-type participant 01 and mutant participant 07, respectively). Electropherograms from ABCG2 gene sequencing showed part of the fragment of interest. The yellow arrows in Figure 2 indicate nucleotides C (forward primer) and G (reverse primer), representing the 421 C/C: wild-type genotype. The yellow arrows in Figure 3 indicate nucleotides A (primer forward) and T (primer reverse), representing the genotype 421 A/A: mutant.

The population under investigation had a greater prevalence of the 421 C/C genotype. Additionally, there was a notably reduced occurrence of the 421 A/A genotype in the fasting conditions or carriers with the A/A genotype could not be identified in the fed conditions.

The genetic variations under analysis were found to be in Hardy-Weinberg equilibrium, as determined by a Pearson chi-squared test with a P-value greater than or equal to 0.05. A total of 117 participants were genotyped for the *ABCG2* C421A mutation in both fasting and fed settings. The frequencies of the genotypes are shown in Table 3.

**Table 3:** Genotype frequencies of enzyme transporter genes in the studied population (n=148) under conditions of fasting and fed.

Genotype	Absolute frequency	Genotype frequency	Allele frequency	χ^2^
Fasting (n=60)				
CC	53	0.883	0.925 (p)	0.983
CA	5	0.083		
AA	2	0.033	0.075 (q)	
Fed (n=57)				
CC	53	0.930	0.917 (p)	0.989
CA	4	0.070		
AA	0	0	0.066 (q)	

p: frequency of allele C; q: frequency of allele A. P-value >0.05 (chi-squared test).

### Influence of genetic polymorphism on pharmacokinetics

The means and standard deviations of pharmacokinetic characteristics of rivaroxaban for the different genotypes of the investigated polymorphism are shown in Table 4.

**Table 4:** Pharmacokinetic parameters of rivaroxaban according to *ABCG2* genotype.

Genotype	Fasting (n=60)			Fed (n=57)	
*ABCG2* C421A	CC(n=53)	CA(n=5)	AA(n=2)	CC(n=53)	CA(n=4)
Pharmacokinetic parameters					
C_max_ (ng/mL)	190.19±56.57	176.27±48.18	145.81±17.06	308.06±70.04	360.57±76.48
AUC^0-t^ (ng/mL·h)	1570.77±426.64	1374.69±391.11	1193.81± 13.36	1913.44±480.09	2087.52±428.50
t1/2 (h)	15.47±4.95	16.43±2.52	41.04±23.73*	9.51±4.14	10.23±4.84
Cl /F (mL/h)	13.01±3.34	14.50±4.47	11.82±2.13	11.08±2.95	9.71±1.74
Vd/F (L)	275.59±82.23	334.45±115.23	508.27±72.79*	144.18±60.51	128.62±40.35

Data are reported as mean±SD. AUC^0-t^: area under the concentration-time curve from 0 to 72 h; C_max_: peak concentration; t1/2: terminal half-life; Cl/F: oral clearance; Vd/F: distribution volume. *P<0.05 between genotypes. For two groups, Student's *t*-test was used for comparison; ANOVA was used to compare the means of three groups.

**Figure 4:**
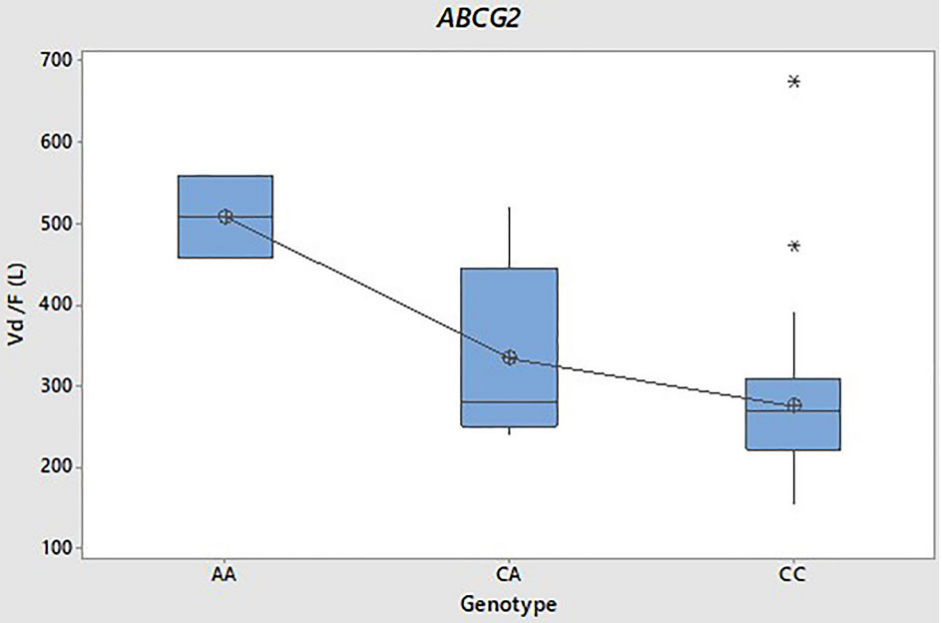
Distribution volume (Vd/F) (L) box plot of rivaroxaban under fasting conditions for *ABCG2* gene 421C>A genotypes. Boxes include the median, quartile (Q)1 and Q3, and whiskers indicate the maximum and minimum values. *Outliers.

**Figure 5:**
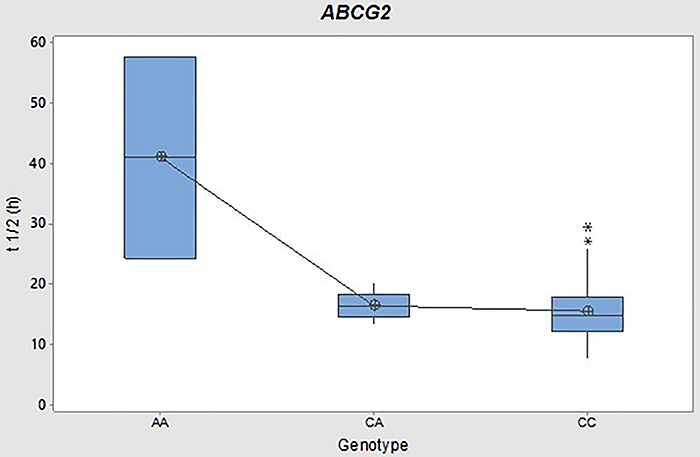
Box plot of terminal half-life (t1/2) (h) of rivaroxaban under fasting conditions for *ABCG2* gene 421C>A genotypes. Boxes include the median, quartile (Q)1, and Q3, and whiskers indicate maximum and minimum values. *Outliers.

Individuals carrying two mutant alleles in the *ABCG2* SNP tended to have lower AUC, Cmax, and Cl/F (as shown in Table 4) compared to carriers of the wild-type genotype. However, it is essential to note that these differences did not achieve statistical significance (P>0.05). In our dataset, individuals with the *ABCG2* 421 A/A genotype had considerably higher average values of Vd/F (508.27±72.79 L; P=0.001) and t1/2 (41.04±23.73 h; P=0.000) compared to those with the other genotypes, 421 C/A and 421 C/C, with no statistically significant difference between means. Figures 4 and 5 are box plots of the Vd/F and t1/2 of the fasting conditions, respectively, categorized by genotype.

## Discussion

DOACs have been associated with various adverse drug reactions (ADRs), especially hemorrhagic events. As a result, many authors and medical specialists have expressed the need for therapeutic monitoring of DOACs. This has led to pharmacogenetic markers being explored as a potential option for customized therapy in a large patient population (12,14,17). Pharmacogenetics is beneficial for personalizing and modifying pharmacological interventions, leading to enhanced effectiveness and tolerability. Accordingly, it has the potential to refine the rivaroxaban prescription process (13-15,19,20). The findings of this investigation indicated that *ABCG2* genotypes may impact the pharmacokinetics of rivaroxaban in individuals in good physical condition.

The *ABCG2* 421C>A polymorphism is considered the most significant, as it has been the most studied polymorphism to date and has a low allele frequency of more than 10% of the global population. This polymorphism in the *ABCG2* gene has been found to affect the activity of the BCRP transport protein and consequently lead to a reduced elimination of substrate drugs and subsequently increased concentration. It has been shown that individuals carrying the A allele tend to exhibit diminished function of the transportation of medications that are substrates for BCRP. A higher level of drug substrates is associated with a higher risk of ADRs (15-19,21).

The BDDCS allows some general predictions regarding the role of transporters in oral drug disposition based on the drug's classification. Class 2 compounds can be effluxed out of enterocytes and are subject to efflux transporter effects that can influence bioavailability and absorption rates. Due to the low solubility limiting luminal concentration, they are unlikely to saturate efflux transporters (23). This research found that the two individuals carrying the 421 A/A genotype had a tendency to present a lower mean (Table 4) for Cmax; however, the difference was not statistically significant (P>0.05). While it was found that the polymorphism in the *ABCG2* gene may influence the absorption rate (Cmax), the small number of participants carrying this genotype must be considered when interpreting this finding. Therefore, inhibition of BCRP is likely to result in decreases in Cmax, as the efflux transporter supposed to be involved in rivaroxaban disposition can no longer take rivaroxaban from the enterocytes and into the intestinal lumen, thereby decreasing the absorption rate (10).

Furthermore, in our data, carriers of the *ABCG2* A/A genotype presented significantly higher mean values of Vd/F (508.27±72.79 L; P=0.001) and t1/2 (41.04±23.73 h; P=0.000) than the other genotypes, 421 C/A and 421 C/C, which did not have a statistically significant difference between their means.

The resulting change in volume of distribution can lead to a significant change in drug effect or likelihood of toxicity, as well as a change in half-life, regardless of a change in clearance. Efflux transporters can be inhibited, affecting drug transport from renal epithelial cells into the urine. Therefore, the drug is better distributed in the body and there is less drug in the systemic circulation, making it less available to be cleared by the kidneys. These factors contribute to a longer half-life. In this study, the changes in volume and clearance were not correlated. While altering the function of hepatic apical efflux transporters may influence Vd/F, this effect is not always predictable. Decreasing hepatic basolateral efflux may keep the drug within hepatocytes, possibly reducing plasma levels and increasing Vd/F. Therefore, it seems that efflux transporter inhibition leads to a decrease in the distribution volume for the central compartment and an increase in the distribution volume for the peripheral tissue compartments. Accordingly, an increase in total distribution volume is evident when there is a kidney interaction (11).

Currently, only one pharmacogenetic study has examined the correlation between the genetic polymorphisms of CYP enzymes and ABCB1 and ABCG2 transporters using low concentrations of rivaroxaban. However, the publication did not discuss the findings obtained for the 421C>A polymorphism of the *ABCG2* gene. Nevertheless, a notable observation was made about people who carried the AA alleles, as evidenced by the genotyping of four of the 86 participants. Specifically, these individuals exhibited lower minimum concentrations of rivaroxaban compared to the other patients (12). In our investigation, we assessed the influence of these polymorphisms on five pharmacokinetic parameters. Both investigations showed that AA alleles may impact pharmacokinetic parameters and bioavailability, which suggests that the AA variation is associated with a loss of function.

The pharmacokinetics, pharmacodynamics, and tolerability of medications can vary in patients undergoing long-term therapy, particularly those with underlying medical conditions and taking other medications concurrently. Nevertheless, a study with a single administration of rivaroxaban in individuals without any underlying health issues can still effectively assess the impact of genetic variations on the drug's efficacy. This approach reduces potential confounding variables, such as smoking, controlled dietary circumstances, comorbidities, and concurrent medication usage, among other considerations.

The sample size calculation in this study was not based on the comparison of genotypes, but on bioequivalence assessment. It is crucial to exercise caution when interpreting the results of this pharmacogenetic investigation due to the limited sample size. Generally, it is recommended to rely on observations from multicenter studies that involve a substantial sample size to comprehend the influence of ABC transporter genes on the pharmacokinetics of rivaroxaban. This approach would enable the inclusion of research that includes participants with genotypes of low prevalence.

Rivaroxaban is a DOAC that has been increasingly utilized in the realm of antithrombotic treatments in clinical practice. However, at present, there is a lack of pharmacogenetic recommendations for rivaroxaban. More accurate associations of *ABCG2* genotypes with the expression and function of BCRP protein and more precise dose adjustments are the areas that warrant future study to develop precise medicine strategies related to *ABCG2* genotypes. This approach allows improving anticoagulation treatment in an expanding population of patients undergoing therapy with DOACs.
